# Emergence and Molecular Epidemiology of Human Metapneumovirus in Greece in the Post-COVID Era

**DOI:** 10.3390/ijms27062821

**Published:** 2026-03-20

**Authors:** Nikolaos Siafakas, Korina Papadopoulou, Anastasia Psina, Sotirios Tsiodras, Spyridon Pournaras

**Affiliations:** 1 Clinical Microbiology Laboratory, Attikon University Hospital, 12462 Athens, Greece; korinapap210@gmail.com (K.P.); spournaras@med.uoa.gr (S.P.); 2 Department of Microbiology, Konstantopouleio General Hospital “Agia Olga”, 14233 Athens, Greece; ansts75@gmail.com; 3 4th Department of Internal Medicine, Attikon University Hospital, 12462 Athens, Greece; tsiodras@med.uoa.gr

**Keywords:** human metapneumovirus, genotypic identification, molecular epidemiology, South Greece, *G* 111nt duplication, A2.2.2 and B2 genotypes

## Abstract

Recent reports have advocated the global resurgence of human metapneumovirus (hMPV) infections in the post-COVID-19 era. Considering the absence of relevant data from Greece, the present study aimed to explore the molecular epidemiology and possible resurgence of significant genotypic variants. Whole genome and F gene-specific sequencing were implemented in order to obtain complete information on the genotype and lineage distribution of circulating hMPV strains in Greece. The results showed a statistically significant increase in monthly positivity rates in 2025, especially from February to April, compared to the respective period in 2024. Overall, 21 strains were classified as genotype A2.2.2 (42.9%) and 27 as genotype B2 (55.1%), whereas only one strain belonged to genotype B1. The *G* gene of most completely sequenced A2.2.2 strains harbored a 111nt duplication sequence and a genotype-specific pattern of *N*-glycosylation sites. Maximum likelihood and time-scaled, Bayesian phylogenetic analyses demonstrated the dominance of specific sub-lineages at the regional level and international transmission events. This complex epidemic pattern in conjunction with the differential evolutionary pressure exerted on the hMPV genes, advocates continuous surveillance of hMPV epidemiology with multiple genes, or complete genome sequencing methodologies.

## 1. Introduction

Human metapneumovirus (hMPV) is a significant example of how modern molecular techniques have revolutionized the diagnosis and study of viral pathogens. The virus was retrospectively discovered in 2001 in the supernatants of cell cultures inoculated with respiratory tract samples collected over a period of two decades [1,2]. Following its initial recognition and improved diagnostic capabilities, more data accumulated, including previously undiagnosed cases, and hMPV has gradually gained clinical recognition as a major cause of acute respiratory tract infections worldwide, affecting all age groups [3]. Clinical manifestations range from mild upper respiratory tract infections to severe lower respiratory diseases, such as bronchiolitis and pneumonia, especially in young children, older adults, and immunocompromised individuals [4,5,6]. Re-infections are common throughout life, reflecting incomplete or declining immunity to hMPV [7].

hMPV is an enveloped virus that belongs to the family *Pneumoviridae*. It has a single-stranded, negative-sense RNA genome that is approximately 13.3 kb long and encodes multiple structural and non-structural proteins, among which the fusion (*F*) and attachment (*G*) glycoproteins play central roles in viral entry, host interaction, and immune recognition [8]. Both genes, either in combination or individually, have been extensively used to study the diversity and evolution of circulating hMPV strains. The *F* gene has been widely used for hMPV genotyping and is highly useful for molecular epidemiology studies, because it combines a high degree of sequence conservation and sufficient genetic variability for reliable lineage discrimination, allowing for both robust amplification and phylogenetic analysis [9]. Moreover, the more genetically variable G gene is considered a major driver of antigenic variation and therefore may offer more detailed discriminatory power for tracing hMPV genetic diversity and global transmission patterns [10,11,12,13].

The implementation of novel next-generation sequencing (NGS) methods and the development of highly efficient bioinformatics tools over the past decade have substantially revolutionized our knowledge of hMPV evolution and global circulation. hMPV is classified into two main lineages, A and B, based on *F* and *G* sequences. These are further subdivided into several sub-lineages that have been associated with temporal and regional variations in the epidemiology of hMPV, which has enhanced our understanding of hMPV evolution [14]. Multiple studies have reported events of dynamic lineage turnover, dominance of specific sub-lineages at a regional level, and frequent international transmission events [9,15,16,17]. In particular, contemporary A2-derived sub-lineages, including A2.2.2, have attracted increasing attention because of their widespread dissemination and association with recent outbreaks in different geographic regions [18]. 

Furthermore, a distinctive molecular feature associated with hMPV evolution over the last 10 years is the emergence of insertion and duplication events within the *G* gene of the A genotype strains. Specifically, 180-nucleotide and 111-nucleotide insertions were initially reported for A2.2.1 strains in Japan in 2016 and 2017, respectively [11,12]. Since then, they have been predominantly associated with recent A2.2.2 genotype lineages and have been continuously reported in several countries [9,18,19,20,21]. However, the biological consequences of these duplications are not well understood. Given the emergence and increased prevalence of these variants, they are thought to positively influence viral fitness through mechanisms such as altered glycosylation and antigenic variation, which may contribute to immune evasion and/or modulation of host–virus interactions. These findings underscore the importance of continuous molecular surveillance to track the ongoing evolution of viral populations and to support public health preparedness.

Despite the knowledge that has gradually accumulated over the last ten years regarding the clinical significance and global predominance stemming from a dynamic evolutionary profile, molecular epidemiological data on hMPV in Greece remain scarce. Moreover, there has been increasing concern about the global resurgence of hMPV infections in the post-COVID-19 era, with notable shifts in epidemiology, unusual seasonal patterns, increased co-infection rates, and altered age distributions [22]. In this context, the present study aimed to explore the molecular epidemiology of hMPV strains circulating in Greece during the first few months of 2025. Whole genome sequencing with NGS technology and more conventional F gene-specific sequencing were implemented to (i) characterize the genotype and lineage distribution of circulating hMPV strains in Greece during the post-COVID-19 era, (ii) investigate the possible resurgence of hMPV in the post-COVID era that may be linked to recently emerged genotypic variants, and (iii) assess the presence of contemporary molecular signatures, such as *G* gene duplication variants, which may be linked to increased transmissibility and virulence.

## 2. Results

### 2.1. Identification of hMPV Genotypes

**Figure 1:**
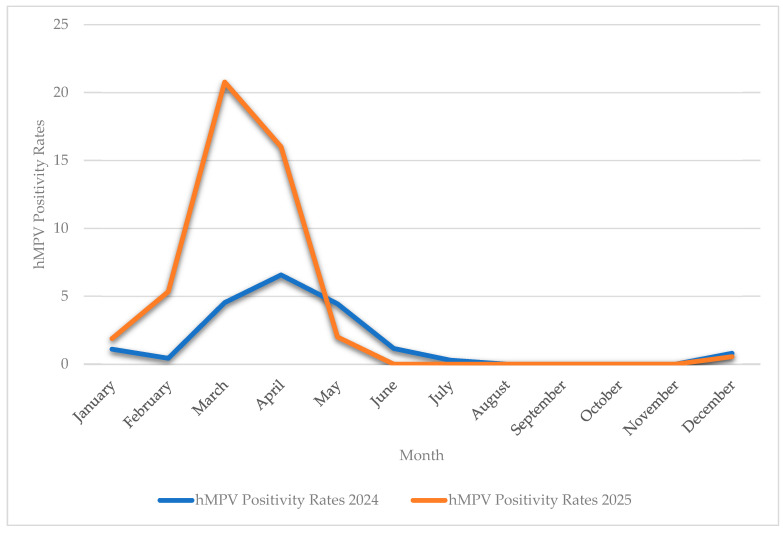
Monthly hMPV positivity rates in 2024 and 2025. These values were calculated as the number of hMPV-positive samples divided by the total number of respiratory specimens tested per month.

Figure 1 illustrates a comparison of monthly hMPV positivity rates between 2024 and 2025. A clear seasonal pattern of hMPV infection was observed in both years, with peak activity observed during late winter and early spring. However, there was a statistically significant increase in monthly positivity rates in 2025, especially from February to April, which culminated in March (incidence rate ratio [IRR] adjusted for calendar month: IRR = 2.49, 95% CI 1.69–3.68, *p* < 0.001).

hMPV was detected in combination with other respiratory viruses in 11.4% and 17.9% of recorded hMPV-positive infections in 2024 and 2025, respectively. The difference in co-infection rates between the two years was not statistically significant (χ^2^ test, *p* > 0.05) (the corresponding numerical data are provided in Supplementary Table S4). Rhinovirus/enterovirus was the most commonly co-detected pathogen in both years. hMPV infections were observed across all age groups in both years, with no statistically significant differences between 2024 and 2025 when the age-group distributions of hMPV infections were compared (χ^2^ test, *p* > 0.05) (relevant details are shown in Supplementary Table S5).

Table 1 presents all hMPV genotypes identified in the present study, along with relevant data including patient demographic details, specimen type and date of collection, syndromic testing results, method used, genomic sequence coverage and *G* gene duplication segments for A2.2.2 genotype strains. A complete genome sequence was recovered from 27 of the 29 specimens analyzed by NGS, with partial *G* and/or *F* sequences recovered from the remaining two samples. Sequencing metrics extracted from the DRAGEN analysis reports indicated high-quality sequencing libraries with Q scores ranging from approximately 32 to 33. Genome coverage for most samples exceeded 99%, with median sequencing depths ranging from tens to several thousand reads per nucleotide position. Overall, 21 strains were classified as genotype A2.2.2 (42.9%), 27 as genotype B2 (55.1%), and only one strain belonged to genotype B1. Both the complete genome sequences and partial *F*/*G* gene sequences of the hMPV strains detected in the present study were highly similar (>99%) to the respective sequences of hMPV strains that circulated worldwide in outbreaks and sporadic cases.

### 2.2. Genetic Analysis of the hMPV Strains

**Figure 2:**
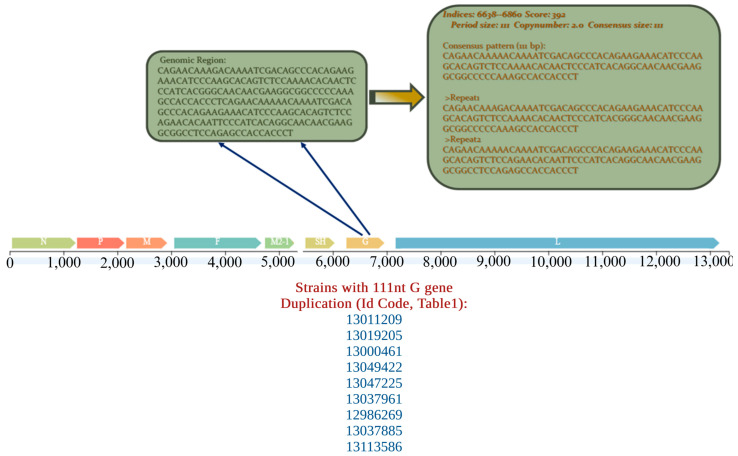
One 111nt *G* gene duplication found in 9 A2.2.2 genotype strains of the present study.

First, no genetic recombination events were detected in the complete genome sequences of the 27 hMPV strains; however, one 111nt repeat in the *G* gene sequence of nine out of the 12 strains belonging to A2.2.2 genotype was identified (Figure 2), whereas neither a 111nt, nor a 180nt duplication in the *G* gene was observed in the remaining strains.

**Figure 3:**
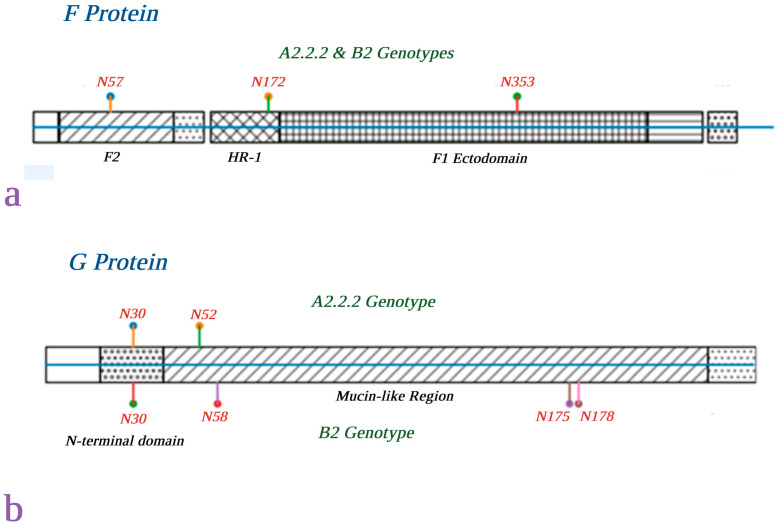
Predicted glycosylation sites on the *F* (**a**) and *G* (**b**) protein sequences of the hMPV strains.

In silico analysis of the complete *F* and *G* gene sequences obtained from the whole genome sequences of the 27 hMPV strains, predicted N-linked glycosylation motifs (Asparagine-X-Serine/Threonine, or N-X-S/T, where X is any amino acid except proline) were identified in the respective amino acid sequences of the fusion (*F*) and attachment (*G*) glycoproteins. Three N-linked glycosylation motifs were completely conserved in the *F* protein across all strains, irrespective of the genotype or year of detection. These motifs were located at positions N57, N172, and N353 and belonged to specific functional domains of the *F* protein, including the F2 subunit, heptad repeat 1 (HR-1) region and F1 ectodomain, respectively [23] (Figure 3a). In contrast, potential glycosylation motifs in the *G* protein exhibited different, genotype-specific patterns. Specifically, all A2.2.2 strains consistently carried two N-linked glycosylation motifs (N30 and N52), whereas most B2 strains predominantly had four motifs (N30, N58, N175, and N178) [24] (Figure 3b), with the exception of one B2 strain (480979/B2/ATH/2025), which exhibited a slight displacement of two motifs (N180 and N183, instead of N175 and N178, respectively). The N30 motif was located within the N-terminal domain, whereas the remaining motifs were located within the extracellular mucin-like domain of the *G* protein.

### 2.3. Comparative Phylogenetic Analysis of the hMPV Strains

**Figure 4:**
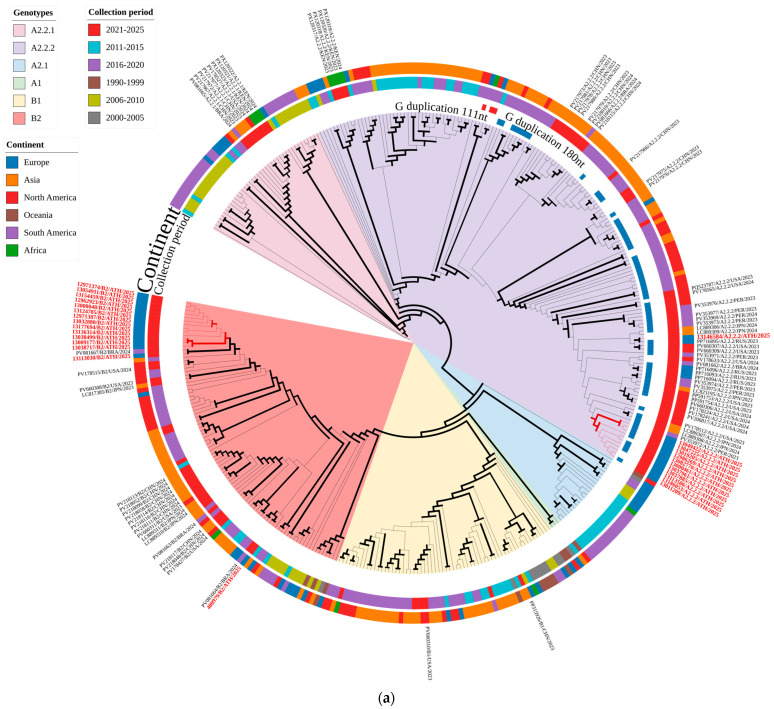

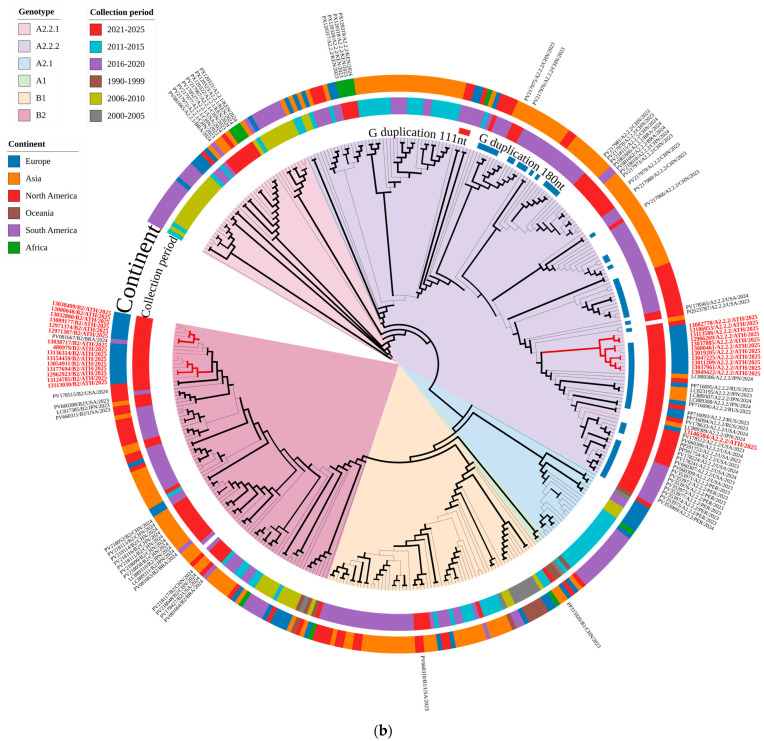
Maximum Likelihood dendrogram showing the phylogenetic comparison of strains belonging to different genotypes that have circulated worldwide during the past few decades, on the basis of *F* (**a**) and *G* (**b**) gene sequences, respectively. The 27 hMPV strains detected in Greece and analyzed in the present study using whole-genome sequencing are highlighted in red. Bold branch lines indicate clades supported by >70% bootstrap values. Only the names of strains detected in the post-COVID era from 2023 onwards are shown. Details of all the strains used for the dendrograms are provided in Supplementary File S1 (Tables S1 and S2).

Figure 4 shows comparative phylogenetic analyses of strains belonging to different genotypes that have circulated worldwide over the past few decades, based on *F* (a) and *G* (b) gene sequences, together with the hMPV strains that were genotyped in the present study. Clear separation of the different genotypes was evident for both gene sequences and was supported by high bootstrap values. A preponderance of A2.2.2 strains carrying the 111nt duplication in their *G* gene was also evident, whereas only three of the A2.2.2 strains used for the construction of the Maximum Likelihood dendrograms that circulated in the past had a 180nt duplication. Most strains carrying the duplication were classified into the same sub-clusters supported by high bootstrap values based on both *F* and *G* gene sequences; however, there were also instances in which strains that did not exhibit the duplication were included in those sub-clusters.

Based on the *F* gene sequences (Figure 4a), most A2.2.2 genotype strains detected in the present study formed a distinct cluster, supported by a significant bootstrap value. However, one strain, 13146584/A2.2.2/ATH/2025, clustered separately, along with a strain isolated in Russia in 2023 (GenBank accession no PP716095). In general, all A2.2.2 genotype strains in the present study clustered within a broader clade that included strains isolated in the USA, Peru, Japan and Russia since 2021, as evident from the Maximum Likelihood dendrogram of Figure 4a. In contrast, they clustered separately from strains detected in China in 2023 and 2024.

All but one genotype B2 strain detected in the present study formed a distinct genetic cluster (Figure 4a). These were more closely related to the two strains detected in Brazil (GenBank accession no PV081667) and China (GenBank accession No. PV217945) in 2024 and 2019, respectively. Strain 480979/B2/ATH/2025 formed a separate cluster with 2 strains detected in the Netherlands in 2016 (GenBank accession nos OL794377 and OL794380) and one strain from Brazil, detected in 2024 (GenBank accession no PV081664).

Phylogenetic relationships based on the *G* gene (Figure 4b) also showed a clear distinction between the different genotypes, supported by high bootstrap values, with all strains studied classified as either genotype A2.2.2 or genotype B2. More statistically robust genetic sub-clusters of *G* sequences were supported by a high bootstrap value were observed, compared with the *F* sequences of the same. A similar pattern of genetic clustering for genotype A2.2.2 strains detected in the present study compared with that based on *F* gene sequences, was observed. Strain 13146584/A2.2.2/ATH/2025 was again isolated from the rest of the Greek isolates of the same genotype. It has been more closely classified with strains detected in Japan, the USA and Russia since 2021. The remaining A2.2.2 strains formed distinct sub-clusters, supported by a high bootstrap value. All genotype B2 strains in the present study, including 480979/B2/ATH/2025, formed a distinct sub-cluster, together with a strain detected in Brazil in 2024 (GenBank accession No. PV081667).

### 2.4. Time-Scaled Phylogenetic Analysis of hMPV A and B Genotypes

**Figure 5:**
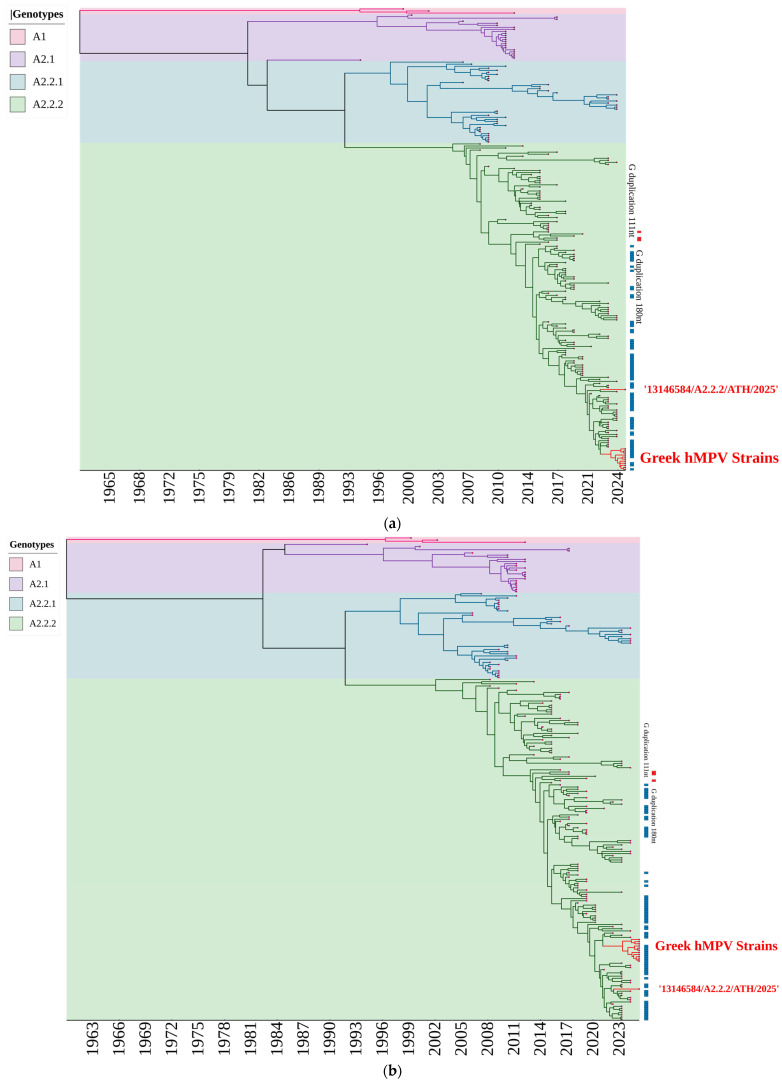

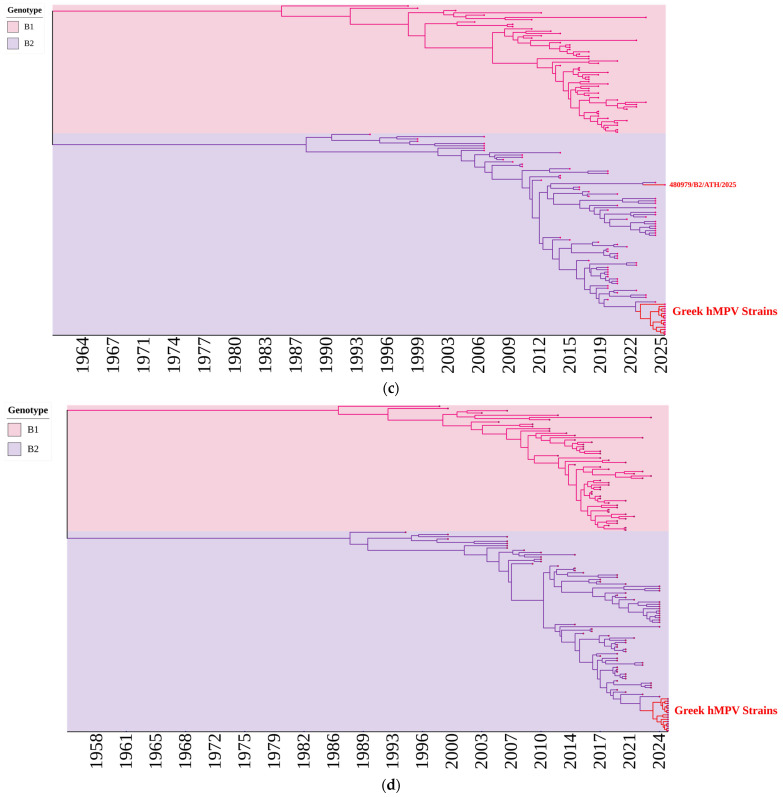
Time-scaled, Bayesian MCMC phylogenetic trees of the hMPV strains analyzed in the present study, along with representative strains from all genotypes that have circulated worldwide during the last few decades. Separate trees were constructed for the *F* genes of genotype A strains (**a**), *G* genes of genotype A strains (**b**), F genes of genotype B strains (**c**) and *G* genes of genotype B strains (**d**). The Greek hMPV strains analyzed in this study are highlighted in red. Details of the hMPV strains are shown in Supplementary File S1, Tables S1 and S2.

The results of the time-resolved Bayesian phylogenetic analyses based on *F* and *G* gene sequences for genotypes A and B are shown in Figure 5. Many sequences have become available worldwide since the implementation of modern molecular detection and sequencing methodologies, which is evident from the clustering of the majority of strains towards later years.

Bayesian phylogenetic reconstruction of genotypes A and B, based either on the *F* gene or *G* gene sequences, verified a distinct genetic separation of the different genotypes (B1, B2, A2.1, A2.2.1, and A2.2.2). The genetic clusters of A1 and A2.1 genotypes ceased in 2012 and 2017, respectively, whereas genotypes A2.2.1 and A2.2.2 were more evolutionarily successful and spanned a longer time period until present. A clear separation between the historical B1 lineage and the more recently dominant B2 lineage is also evident, with B1 strains restricted to earlier periods. Intragenotype clusters of certain sub-lineages that emerged and disappeared abruptly, or circulated until present, were observed for genotypes A2.2.1, A2.2.2 and B2. Most of them exhibited the same temporal signal, corresponding to genetically distant strains that perhaps circulated concurrently but possibly in different geographic regions.

Figure 5a,b also show the gradual preponderance of genotype A2.2.2 strains with the *G* gene 111nt duplication since 2016. In contrast, only three A2.2.2 strains out of the 225 of the same genotype that were used for the construction of the Bayesian, time-scaled phylogenetic trees had the *G* gene 180nt duplication; these three strains were detected in 2016 and 2017 only. The Bayesian, time-scaled phylogenetic reconstruction of the Greek strains yielded a highly concordant topology with that created by the M-L analysis for both A2.2.2 and B2 genotypes; strain 13146584/A2.2.2/ATH/2025 deviated phylogenetically from the rest of the A2.2.2 genotype strains of the present study based on both *F* & *G* gene sequences (Figure 5a,b), whereas strain 480979/B2/ATH/2025 was classified either separately from, or together with the genotype B2 strains of the present study on the basis of the *F* and *G* gene sequences, respectively (Figure 5c,d).

## 3. Discussion

The present study provides the first comprehensive investigation of the molecular epidemiology of hMPV strains in Greece over the past 10 years. The implementation of novel molecular detection methods and sequencing technologies in the present study enabled a more complete analysis of contemporary hMPV strains with regard to their genomic features, genotype distribution, and comparison with global trends in hMPV emergence and epidemiology, especially in the post-COVID era.

There has been increasing concern about the resurgence of hMPV infections in the post-COVID-19 era after the lifting of public health restrictions, accompanied by unusual seasonal patterns, increased co-infection rates and altered age distributions [22,25,26,27,28]. In the present study, a statistically significant rise in hMPV detection rates was recorded for 2025 compared to 2024. In contrast, the expected winter-spring seasonality was preserved and no unusual seasonal patterns were observed. Moreover, trends toward higher co-infection rates and broader age involvement did not reach statistical significance. However, the small number of hMPV-positive cases (n = 62 in 2025 and n = 72 in 2024) provided correspondingly limited statistical power to highlight increased transmission rates as the dominant trend in the post-COVID-19 era of hMPV infection in the regional setting of the present study. Larger datasets spanning all seasons are required to reach statistically significant results in the present study that might corroborate the overall variable regional expression of hMPV epidemiology, according to geographical, climatic, and demographic factors [29,30].

Genotypic identification of hMPV strains in the present study revealed an almost equal representation of the B2 and A2.2.2 genotypes. Several recent studies have shown a gradual rise in A2.2.2 strains, even surpassing B2 strains [9,20,31]. However, to date, the predominance of a single genotype is usually reported as nothing more than a regional/temporal shift (often post-2016 and, especially, post-2021 in some regions) [19]. In contrast, other studies have reported the post-pandemic prevalence of B1 and B2 genotypes, in contrast to the dominance of A2.1, A2.2.1, and A2.2.2 before the pandemic [22]. Nevertheless, it has been suggested that hMPV genotype A evolves faster than genotype B [18], which is also evident from the variety of sub-lineages that belong to genotype A. The time-scaled, Bayesian MCMC phylogenetic trees in Figure 5 clearly show the four different A genotypes that have been characterized since the discovery of hMPVs, in contrast to the only two B genotypes.

Nine out of the 11 A2.2.2 strains that were completely sequenced in the present study had the 111nt duplication in the *G* gene. This duplication event has emerged as the predominant type of A2.2.2 genotype strains in several countries during the post-pandemic era [9,18,19,20,32,33,34,35]. *G*-gene variants carrying the 111-nt nucleotide duplication have been reported in both the A2.2.1 and A2.2.2 lineages of human metapneumovirus; however, a distinct evolutionary mechanism has been suggested for each of these genotypes. An 180nt duplication most likely emerged first in 2011 in A2.2.1 genotype strains, and the 111nt duplication strains appeared around 2014, possibly from an independent secondary duplication event within strains already carrying a 180-nt duplication [11]. The 180nt duplication was first reported in genotype A2.2.2 strains more than ten years ago [36]. However, in this genotype, the 111nt duplication variants diverged from the 180nt duplication clade via a 69-nucleotide deletion event within the duplicated regions of the 180nt variant, rather than through independent duplication events that have been proposed for the A2.2.1 genotype strain [11,12,18].

The A2.2.2 hMPV lineage with *G* gene duplication has been linked with better viral fitness, as they exhibit faster evolutionary rates and eventually achieved higher predominance in shorter periods of time, in comparison with older lineages [20]. However, is the A2.2.2 genotype and its distinct genetic features (*G* gene duplication) linked with increased transmissibility and virulence? Which biological features confer an evolutionary advantage on these strains? It has been suggested that duplication-bearing A2.2.2 variants achieve epidemiological success primarily through enhanced transmissibility and immune escape, and there is no evidence to date that links these dominant variants with increased virulence and a more severe clinical impact. Specifically, these gene duplications are located within the extracellular, mucin-like domain of the *G* glycoprotein and have been suggested to induce protruding subunits that enhance altered glycosylation patterns, increased shielding of antigenic epitopes and antigenic variability, subsequently leading to immune evasion and modulation of host–virus interactions [11,12,37,38]. It was interesting to observe, however, that some A2.2.2 strains, including one strain in the present study, lacking a *G* gene duplication have phylogenetically clustered together with 111nt variants on the basis of the *G* gene sequence (Figure 4b and Figure 5b). This is due to the fact that the *G* gene duplication event is not in itself a marker that defines phylogeny, but rather a variable characteristic within genotype A2.2.2 [12,19].

A larger protruding subunit caused by the 180nt duplication may indicate that the 180nt *G* gene duplication confers a better immune evasion mechanism and is, thus, actually the more evolutionarily successful and subsequently predominant lineage. However, the A2.2.2 lineage with the 111nt *G* gene duplication has emerged as the predominant one, as shown by the Bayesian MCMC phylogenetic trees in Figure 5a,b. This may be explained by the indirect interference of a larger *G* gene duplication with *F*-mediated membrane fusion and spatial organization of envelope glycoproteins via steric hindrance, ultimately reducing overall viral fitness compared with variants carrying the shorter 111nt *G* gene duplication [20,37].

Determination of the predicted N-linked glycosylation motifs in the fusion (*F*) and attachment (*G*) glycoproteins of the strains analyzed by complete genome sequencing in the present study revealed a clear contrast between the genes encoding these two proteins. On the one hand there was complete conservation of three glycosylation sites in the *F* protein (N57, N172, and N353), irrespective of genotype, which indicates the evolutionary pressure for structural/functional conservation of this protein, as has been reported previously [39,40,41]. In contrast, a genotype-specific glycosylation pattern was observed in *G* protein, predominantly within the extracellular mucin-like domain. This is consistent with the genetic variability of the attachment protein, which underlies its structural flexibility and variability in the number and distribution of glycosylation motifs [38,42], likely reflecting a response to the greater, immune-driven selectional pressure acting on this protein.

The detection of a single genotype B1 strain during the present study was unexpected, considering that B1 has been largely displaced by B2 globally [19]. However, sporadic detection of B1 has recently been reported in several studies, suggesting that shifts in dominance rather than lineage extinction has so far characterized genotype B molecular epidemiology [43,44]. Another important observation was the phylogenetic separation of one B2 strain (480979/B2/ATH/2025) from the rest of the Greek B2 strains based on the F gene sequences, while all strains clustered together in their *G* gene sequences (Figure 4a,b and Figure 5c,d). Unfortunately, detailed epidemiological data regarding a distinct travel history, unique clinical presentation, or different outcome compared to other B2 cases were unavailable for the patient infected with the specific strain. Nevertheless, this may reflect the differential, gene-specific evolutionary pressures exerted on the hMPV genome [45]. The *G* gene codes for the attachment protein which is more vulnerable to immune selection and thus, exhibits greater genetic variability, whereas the relative conservation of the *F* gene is linked to less pronounced exposure to immune pressure and the need for structural conservation of the fusion protein and its crucial function in the virus’ life cycle [16,46,47]. Why did we see divergence in a more conserved genomic region and clustering in a more variable? Perhaps the answer lies in the fact that the more variable *G* gene, is more indicative of a convergent sequence similarity amongst epidemiologically related strains, or, in other words, dominance of a certain genetic variant during the acute phase of an outbreak. In contrast, the observed genetic discrepancy in a less variable gene such as *F* may possibly reflect a deeper evolutionary signal of genetic deviations that occurred further back in the past. In summary, these may have been genetically divergent strains that converged to a dominant genetic variant of their attachment proteins due to selective pressure during a certain, regional and temporal epidemic phenomenon. Multiple studies have reported events of dynamic lineage turnover, dominance of specific sub-lineages at a regional level, and frequent international transmission events [9,15,16,17]. These findings highlight the importance of studying the molecular epidemiology of hMPV based on complete genome sequences, or at least, multiple genes, as reliance on a single genomic region, especially a highly variable one, may only represent an acute epidemic event and obscure deeper evolutionary relationships.

## 4. Materials and Methods

### 4.1. Respiratory Specimens and Viral RNA Extraction

A total of 49 respiratory samples that had initially tested positive for hMPV using multiplex syndromic molecular assays over a five-month period (January to April 2025) were retrospectively analyzed in the present study. Co-detection of genetic material from other respiratory viruses, in addition to hMPV, was also observed in respiratory samples from seven patients (two were also positive for rhinovirus–enterovirus, two for respiratory syncytial virus, one for influenza type A H1N1pdm09 virus, one for influenza B virus, and one for adenovirus + rhinovirus–enterovirus). These samples were included consecutively based on the availability of adequate residual material and no additional selection criteria were applied. They were collected from a respective number of patients (29 males and 20 females), with an age range of 49 days to 92 years (15 children and 34 adults), presenting with symptoms of upper and/or lower respiratory tract infection (URTI/LRTI) at ATTIKON University Hospital, in Athens, Greece. They consisted of 39 nasopharyngeal swabs, six bronchoalveolar lavage (BAL) specimens, two sputum samples, and two bronchial aspirates. Nasopharyngeal samples were collected using flocked, synthetic sterile swabs (FLOQSwabs, Copan Italia S.p.A., Brescia, Italy) and immersed in MicroTest^TM^ M4RT 3 mL liquid viral transport medium (Thermo Fisher Scientific Inc., Lenexa, KS, USA). The platforms used for syndromic testing were BioFire^®^ FilmArray^®^ Respiratory Panel 2.1 plus (bioMérieux, Marcy-l’Étoile, France) and QIAstat-Dx Respiratory Panel Plus (QIAGEN, Hilden, Germany) for upper respiratory tract samples (nasopharyngeal swabs), whereas BioFire^®^ FilmArray^®^ Pneumonia Panel (bioMérieux, Marcy-l’Étoile, France) had been employed for lower respiratory tract samples.

To investigate whether there was an upsurge of respiratory hMPV infections in the early part of 2025 in the regional setting of the present study, the rates of hMPV-positive respiratory samples during each calendar month in 2024 and 2025 were compared. All data regarding monthly total tests performed by multiplex syndromic molecular assays and the respective number of total and hMPV-positive samples for 2024 and 2025 are provided in Supplementary Table S3. Positivity rates were calculated as the number of hMPV-positive respiratory samples divided by the total number of respiratory specimens tested by multiplex molecular assays during each calendar month. Seasonal variation was accounted for by fitting a Poisson regression model with log link, including calendar month as a categorical covariate. and estimation of IRR. The logarithm of the total number of tested samples per month was incorporated as an offset term, allowing estimation of incidence rate ratios (IRR) with 95% confidence intervals (CI). A two-sided *p*-value < 0.05 was considered statistically significant. Comparisons of co-detection rates and age-group distributions between 2024 and 2025, were performed using the χ^2^ test.

**Figure 6:**
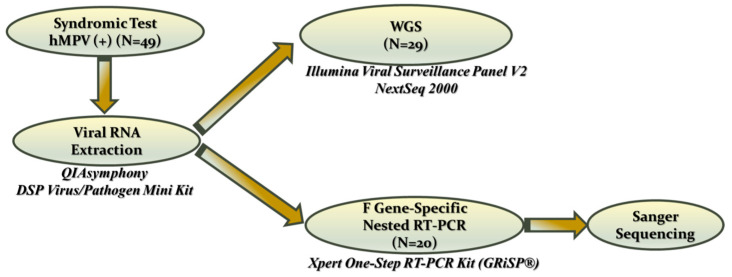
General schematic of the laboratory methods used in the present study.

After testing with either of the aforementioned multiplex syndromic molecular assays, the samples were stored at −80 °C until RNA extraction was performed using the QIAsymphony Virus DSP pathogen mini kit on the QIASymphony SP/AS Platform (QIAGEN, Hilden, Germany), in accordance with the manufacturer’s instructions. All extracted RNA samples were then used either immediately or stored at −80 °C before further processing, as depicted in Figure 6. Whole genome sequencing (WGS) was performed on the first 29 consecutive samples. Owing to resource limitations, the remaining 20 samples were analyzed by conventional Sanger sequencing of the *F* gene to ensure sufficient genotypic characterization.

### 4.2. Whole Genome Sequencing

The complete genome sequences of the hMPV strains detected in 29 samples were subsequently determined using next-generation Sequencing (NGS) technology. Library preparation was performed using the Illumina RNA Prep with Enrichment, Viral Surveillance Panel v2 (Illumina, San Diego, CA, USA), in accordance with the manufacturer’s instructions. This method relies on targeted nucleic acid enrichment designed for the detection and full or near-full genome sequencing of more than 220 different viruses, even in samples with low viral loads, thereby enabling high-resolution characterization of the viral composition and genetic diversity of clinical specimens. Sequencing of the enriched library was performed using an Illumina NextSeq 2000 system (Illumina, San Diego, CA, USA), in accordance with the manufacturer’s instructions. All sequences were submitted to GenBank and their accession numbers are available in the Supplementary File S1 (Supplementary Table S1).

### 4.3. F Gene-Specific Nested RT-PCR

The partial *F* gene sequence of strains detected in 20 samples was amplified using a conventional Nested Reverse Transcription Polymerase Chain Reaction (RT-PCR) protocol with primers designed by Yi et al. [15]. The outer primers were hMPV-F-F1-5′-CAATGCAGGTATAACACCAGCAATATC-3′ and hMPV-F-R1 5′-GCAACAATTGAACTGATCTTCAGGAAAC-3′, whereas the inner primers were hMPV-F-F2-5′-ACATGCCAACATCTGCAGGACAAATAAAAC-3′ and hMPV-F-R2 5′- ACATGCTGTTCACCTTCAACTTTGC-3′. The reverse transcription reaction that converted the extracted RNA into cDNA was performed with the aid of the Engineered M-MLV Reverse Transcriptase Basic Kit (Enzyquest, Heraklion, Greece). First, 20 pmol of the outer reverse and 2 μL of 10 mM deoxynucleotides (dNTPs) were added to 10 μL of extracted RNA and incubated at 65 °C for 10 min, after which they were immediately placed on ice. A reverse transcription reaction mix was then added containing RT Reaction Buffer 5×, 0.01 M DTT, 20 units of ribonuclease inhibitor (Promega Corporation, Madison, WI, USA) and 100 units of Moloney Murine Leukemia Virus reverse transcriptase (M-MuLV RTase), up to a final reaction volume of 20 μL. Reverse transcription was carried out at 42 °C for 50 min, and the M-MuLV RTase was inactivated by heating at 65 °C for 20 min. The produced cDNA was amplified by PCR using a reaction mixture of 50 μL/tube containing 5 μL 10× PCR buffer, 4 μL dNTPs 0.25 mM each, 2 μL MgCl_2_ 25 mM (yielding a final [MgCl_2_] = 2 mM), 25 μL RNase-free water, 2.5 units Taq Polymerase (HotStar Taq DNA Polymerase, Qiagen, Hilden, Germany), 40 pmol of each of the two outer primers and 10 μL cDNA. An initial step of heating at 95 °C for Taq polymerase activation preceded 40 cycles of denaturation (95 °C, 30 s), annealing (65 °C, 30 s), and extension (72 °C, 60 s), followed by a 10 min incubation at 72 °C to complete the extension of the primers. Two microliters of the first PCR product were used as a template for the second, nested PCR using the inner primers and the same thermal cycling conditions and reagent concentrations with the first PCR. The final amplicon that corresponded to a partial fragment of the F gene was approximately 610 bp. It has to be pointed out that, despite occasional, single nucleotide primer mismatches that were observed in some hMPV strains, partial amplification of the *F* gene was consistently successful, as confirmed by the quality of the sequences that were eventually obtained and used for downstream analyses.

The reaction products were visualized by electrophoresis on a 1% low-melting agarose gel (Metaphor FMC Bioproducts, Rockland, ME, USA) stained with 1 μg/mL ethidium bromide. RT-PCR products were excised from the electrophoresis gel using a clean scalpel and purified with the QIAquick gel extraction kit (Qiagen, Hilden, Germany). Both strands were subjected to Sanger sequencing by VBC-Biotech GmbH (Vienna, Austria), using the same set of primers employed in the final round of the nested RT-PCR protocol. All sequences were submitted to GenBank and their accession numbers are available in Supplementary File S1 (Supplementary Table S1).

### 4.4. Bioinformatic Analysis

**Figure 7:**
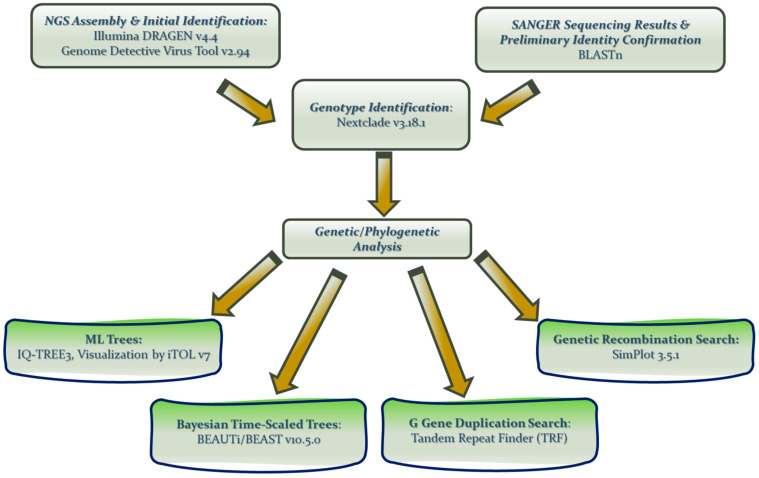
Diagrammatic illustration of the bioinformatics analysis pipeline that was implemented during the present study, from assembly of raw sequence data and initial virus identification to genotype identification and further genetic/phylogenetic analyses.

A specific bioinformatic pipeline was designed for accurate genome reconstruction, reliable strain identification, and robust molecular epidemiology analysis of all sequencing data generated through either whole-genome sequencing or *F* gene-specific conventional Sanger sequencing. Figure 7 shows a schematic overview of the bioinformatics workflow that illustrates the sequential steps from viral genome assembly and initial identification to meticulous genetic and phylogenetic analyses.

#### 4.4.1. Assembly of Whole Genome Sequences and Initial Identification of hMPV Strains

Reconstruction of viral genomes from high-quality raw sequencing data generated from the Illumina NextSeq 2000 platform was performed using both reference-guided and de novo assembly, with the aid of the Viral Surveillance Panel analysis pipeline v4.4 within the Illumina DRAGEN Bio-IT Platform (Illumina Inc., San Diego, CA, USA). Reads with low sequencing quality were automatically removed during the preprocessing step by DRAGEN. High-quality reads were subsequently used for consensus sequence assembly and taxonomic classification through alignment to viral reference genomes included in the Illumina Viral Surveillance Panel database. Consensus genome sequences were generated from mapped reads using a minimum read depth per nucleotide position of 10×, whereas genomic positions with lower coverage were masked as ambiguous bases (N). Genomes with ≥95% genome coverage were considered complete genomes, whereas the remainder were classified as partial genomes. The identity of the assembled viral genomes and the assessment of genome coverage were further confirmed using the Genome Detective Virus Tool (v2.94).

Initial identification and taxonomic confirmation of hMPV strains from either assembled whole-genome sequences or F gene sequences were performed using the BLASTn software against the NCBI nucleotide database (NCBI BLASTn, https://blast.ncbi.nlm.nih.gov/Blast.cgi) (accessed on 15 January 2026) [48].

#### 4.4.2. Genotypic and Phylogenetic Analysis

Genotypes were assigned to all hMPV sequences using the Nextclade/Nextstrain software (v3.21.0) [49]. Nucleotide fragment duplications in the *G* gene sequences were searched for using the Tandem Repeats Finder (TRF), v4.09 [50]. Genetic recombination analysis of the complete hMPV genome sequences was performed with SimPlot, v3.5.1 [51].

Comparative phylogenetic analyses of strains belonging to different genotypes were conducted separately based on F and G gene sequences. The separate datasets for each gene included the hMPV strains detected in the present study, strains that were most closely related to the Greek strains according to analysis with the BLASTn software, and randomly selected, representative strains that currently circulate, or have circulated globally since the late 1990s, and were retrieved from the GenBank/NCBI nucleotide database. Only sequences of high quality and homologous to the genomic regions analyzed in the present study were included in the datasets. Many highly similar sequences from the same location and time period are widely available in the database; therefore, representative strains were selected from each location and time period to minimize sampling bias by ensuring broad temporal and geographic coverage.

In total, the same 378 different hMPV strains, including those of the present study, were used for both *F* gene- and *G* gene-based phylogenetic analyses. All strains were named according to their GenBank accession number first, followed by their genotype, country, and year of detection (e.g., KJ627437/A2.2.1/PER/2011, where PER indicates Peru). An isolate code, instead of a GenBank accession No, is provided for the strains of the present study, where the city of isolation is denoted within the fasta names (ATH for Athens). All information on the strains used is provided in Supplementary Tables S1 and S2.

Multiple sequence alignments were generated and manually inspected using MAFFT v7 [52], prior to tree reconstruction. Maximum likelihood phylogenetic trees were inferred using IQ-TREE v3 [53], and their statistical significance was estimated by calculating the confidence values for the groupings (bootstrap values) with 1000 pseudo-replicates. The best-fit nucleotide substitution models used by IQ-TREE were TIM2 + F + I + G4 for *F* gene sequences and GTR + F + I + R3 for *G* gene sequences. The resulting trees were finally visualized and annotated using the iTOL (Interactive Tree of Life) v7 software.

Estimation of time-scaled phylogeny for the hMPV strains was performed using the Bayesian Markov Chain Monte Carlo (MCMC) method implemented in BEAST software, v1.10.5 [54]. Separate analyses were conducted for the genotype A and B datasets for both *F* and G genes. Regarding the F gene, both genotype datasets were analyzed using MCMC chains of 20,000,000 states, with parameters sampled at steps of 1000. For the *G* gene, the genotype datasets were analyzed using longer MCMC chains of 50,000,000 states to obtain larger effective sample sizes (ESS), reflecting the increased sequence variability of this gene. Convergence and mixing of the MCMC chains were assessed using Tracer v1.7 [55] and the results showed that all analyses reached satisfactory convergence, with ESS values exceeding 200 (*F* gene A genotype: ESS = 369.5; *F* gene B genotype: ESS = 302.3; *G* gene A genotype: ESS = 354.9; and *G* gene B genotype: ESS = 1186.9). A 10% burn-in was applied to each analysis (2,000,000 states for *F* gene datasets and 5,000,000 states for *G* gene datasets) prior to downstream maximum clade credibility (MCC) tree summarization using TreeAnnotator implemented within the BEAST v1.10.5 software package, discarding the first 2000 trees for the *F* gene analyses and the first 5000 trees for the *G* gene analyses as burn-in. The resulting MCC trees were visualized and annotated using FigTree v1.4.5 [56].

## 5. Conclusions

The present study provides comprehensive molecular epidemiological data on circulating hMPV strains in Greece during the post-COVID era. Increased transmission may have constituted the only dominant trend in the post-COVID era of hMPV infections in this regional setting, in contrast to other studies that reported an overall shift in seasonal patterns and patient age distribution. However, larger datasets spanning multiple seasons are required to determine whether broader epidemiological changes, including altered seasonal patterns, shifts in age distributions and co-infection rates, have occurred in Greece during the post-COVID era. The co-circulation of the globally dominant A2.2.2 and B2 lineages was observed, with a high prevalence of A2.2.2 strains carrying the contemporary 111-nt *G* gene duplication. Phylogenetic and time-scaled analyses supported both the regional clustering of most Greek hMPV strains and their interconnection with global transmission networks. Moreover, the discrepant glycosylation patterns of the more variable *G* protein across different genotypes, together with the observed genetic deviation of one B2 strain in the less variable *F* gene, highlight the complex, highly interconnected and dynamically evolving pattern of hMPV circulation. Collectively, these findings signify the necessity for continuous surveillance of hMPV epidemiology based on multiple genes’, or complete genome sequences.

## Figures and Tables

**Table 1:** hMPV genotypes identified during the present study, patient demographic data, specimen type and date of collection, syndromic testing results, method used, genomic sequence coverage and *G* gene duplication segments of A2.2.2 strains. (Abbreviations: M, Male; F, Female; BAL, Bronchoalveolar Lavage; RV-EV, Rhinovirus–Enterovirus; RSV, Respiratory Syncytial Virus; ADV, Adenovirus; WGS, Whole Genome Sequencing; CG, Complete Genome; n/a, not applicable).

Code	Collection Date	Age	Sex	Sample Type	Syndromic Testing Result	Genotype	Method	Genome Coverage	G Gene Duplication
480979	23 January 2025	3	M	Nasopharyngeal Swab	hMPV	B2	WGS	CG	n/a
481108	11 February 2025	35	M	Nasopharyngeal Swab	hMPV	A2.2.2	*F* GENE	PARTIAL *F*	None
12971374	15 January 2025	42	M	Nasopharyngeal Swab	hMPV	B2	WGS	CG	n/a
12971387	15 January 2025	73	F	Nasopharyngeal Swab	hMPV	B2	WGS	CG	n/a
12986269	21 January 2025	65	M	Nasopharyngeal Swab	hMPV	A2.2.2	WGS	CG	***111nt***
13000048	27 January 2025	71	F	Nasopharyngeal Swab	hMPV	B2	WGS	CG	n/a
13000461	28 January 2025	7	F	Nasopharyngeal Swab	hMPV/INFLUENZA B	A2.2.2	WGS	CG	***111nt***
13003711	28 January 2025	76	M	Nasopharyngeal Swab	hMPV	B2	*F* GENE	PARTIAL *F*	n/a
13007671	29 January 2025	40	M	Nasopharyngeal Swab	hMPV	B2	*F* GENE	PARTIAL *F*	n/a
13009177	31 January 2025	38	M	Nasopharyngeal Swab	hMPV	B2	WGS	CG	n/a
13011209	31 January 2025	9	M	Nasopharyngeal Swab	hMPV	A2.2.2	WGS	CG	***111nt***
13012232	1 February 2025	?	M	Nasopharyngeal Swab	hMPV/RV-EV	B2	*F* GENE	PARTIAL *F*	n/a
13019205	4 February 2025	71	F	Nasopharyngeal Swab	hMPV/RSV	A2.2.2	WGS	CG	***111nt***
13032880	10 February 2025	73	M	Nasopharyngeal Swab	hMPV	B2	WGS	CG	n/a
13037885	12 February 2025	8	M	Nasopharyngeal Swab	hMPV	A2.2.2	WGS	CG	***111nt***
13037961	12 February 2025	49	M	Nasopharyngeal Swab	hMPV	A2.2.2	WGS	CG	***111nt***
13038499	12 February 2025	80	M	Nasopharyngeal Swab	hMPV	B2	WGS	CG	n/a
13038717	12 February 2025	48	F	Nasopharyngeal Swab	hMPV	B2	WGS	CG	n/a
13045474	16 February 2025	54	F	Nasopharyngeal Swab	hMPV	B2	*F* GENE	PARTIAL *F*	n/a
13046739	16 February 2025	10	M	Nasopharyngeal Swab	hMPV/RSV	A2.2.2	*F* GENE	PARTIAL *F*	n/a
13047225	16 February 2025	19	M	Nasopharyngeal Swab	hMPV	A2.2.2	WGS	CG	***111nt***
13049422	17 February 2025	49	M	Nasopharyngeal Swab	hMPV	A2.2.2	WGS	CG	***111nt***
13082770	5 March 2025	51	F	BAL	hMPV/INFLUENZA A	A2.2.2	WGS	CG	None
13113586	17 March 2025	10.7 m	F	Nasopharyngeal Swab	hMPV/RV-EV	A2.2.2	WGS	CG	***111nt***
13146854	1 April 2025	8	M	Nasopharyngeal Swab	ADV/hMPV/RV-EV	A2.2.2	WGS	CG	None
13186053	19 April 2025	57	F	Sputum	hMPV	A2.2.2	WGS	CG	None
13113030	17 March 2025	7	F	BAL	hMPV	B2	WGS	CG	n/a
13136314	27 March 2025	44	M	BAL	hMPV	B2	WGS	CG	n/a
13177694	14 April 2025	92	F	Sputum	hMPV	B2	WGS	CG	n/a
13054911	19 February 2025	32	M	Nasopharyngeal Swab	hMPV	B2	WGS	CG	n/a
13124785	21 March 2025	8	F	Nasopharyngeal Swab	hMPV	B2	WGS	CG	n/a
13154459	4 April 2025	56	F	BAL	hMPV	B2	WGS	CG	n/a
12962923	11 January 2025	46	M	Bronchial Aspirate	hMPV	B2	WGS	CG	n/a
13135637	27 March 2025	66	M	BAL	hMPV	B2	WGS	PARTIAL *G*	n/a
12958482	10 January 2025	46	M	Nasopharyngeal Swab	hMPV	B2	*F* GENE	PARTIAL *F*	n/a
12988320	22 January 2025	67	M	Nasopharyngeal Swab	hMPV	B2	*F* GENE	PARTIAL *F*	n/a
13017190	2 April 2025	84	M	Nasopharyngeal Swab	hMPV	A2.2.2	*F* GENE	PARTIAL *F*	n/a
13057877	20 February 2025	66	F	Nasopharyngeal Swab	hMPV	A2.2.2	WGS	PARTIAL *F*&*G*	n/a
13059108	21 February 2025	4	M	Nasopharyngeal Swab	hMPV	B2	*F* GENE	PARTIAL *F*	n/a
13096250	10 March 2025	16	M	Nasopharyngeal Swab	hMPV	B2	*F* GENE	PARTIAL *F*	n/a
13114053	17 March 2025	76	M	Bronchial Aspirate	hMPV	A2.2.2	*F* GENE	PARTIAL *F*	n/a
13117137	18 March 2025	66	F	Nasopharyngeal Swab	hMPV	A2.2.2	*F* GENE	PARTIAL *F*	n/a
13128399	24 March 2025	66	F	BAL	hMPV	A2.2.2	*F* GENE	PARTIAL *F*	n/a
13129850	24 March 2025	3.1 m	F	Nasopharyngeal Swab	hMPV	B2	*F* GENE	PARTIAL *F*	n/a
13149309	2 April 2025	87	F	Nasopharyngeal Swab	hMPV	B2	*F* GENE	PARTIAL *F*	n/a
13157524	6 April 2024	3.7 m	F	Nasopharyngeal Swab	hMPV	A2.2.2	*F* GENE	PARTIAL *F*	n/a
13166891	9 April 2025	69	M	Nasopharyngeal Swab	hMPV	A2.2.2	*F* GENE	PARTIAL *F*	n/a

## Data Availability

All complete, or partial genome sequences of the strains analyzed in the present study are available through Genbank (www.ncbi.nlm.nih.gov/nucleotide) (accessed on 26 January 2026). The GenBank accession numbers are listed in Supplementary File S1. Information about all strains used in the phylogenetic analyses can also be found in Supplementary File S1.

## Supplementary Materials

The following supporting information can be downloaded at: https://www.mdpi.com/article/10.3390/ijms27062821/s1.

## Abbreviations

The following abbreviations are used in this manuscript:

hMPV	Human metapneumovirus
COVID-19	Coronavirus disease 2019
NGS	Next Generation Sequencing
RT-PCR	Reverse Transcription Polymerase Chain Reaction
WGS	Whole Genome Sequencing
nt	Nucleotide
IRR	Incidence Rate Ratio
CI	Confidence Interval
MCMC	Markov Chain Monte Carlo
MCC	Maximum Clade Credibility
ESS	Effective Sample Size
URTI	Upper respiratory tract infection
LRTI	Lower respiratory tract infection
BAL	Bronchoalveolar Lavage
RV/EV	Rhinovirus/Enterovirus
RSV	Respiratory Syncytial Virus
ADV	Adenovirus
BLASTn	Basic Local Alignment Search Tool (nucleotide)
NCBI	National Center for Biotechnology Information
TRF	Tandem Repeats Finder
MAFFT	Multiple Alignment using Fast Fourier Transform
IQ-TREE	Software for inference of Maximum Likelihood Phylogenetic Trees
iTOL	Interactive Tree of Life
BEAST	Bayesian Evolutionary Analysis Sampling Trees
