# Supplementary material for: Emergence and Molecular Epidemiology of Human Metapneumovirus in Greece in the Post-COVID Era
Source: Int J Mol Sci. 2026 Mar 20;27(6):2821. doi: 10.3390/ijms27062821 (PMC13026375; doi:10.3390/ijms27062821)
Supplement: Supplementary file 1 [file ijms-27-02821-s001.zip › ijms-4158257-supplementary.pdf]

Supplementary Table 1. GenBank Accession Nos. of the complete genome, or partial F gene sequences of the strains that were analyzed in the present study.

Isolate	GenBank Accession Number
**481108-ATH-A2.2.2-2025**	PX921703
**13012232-ATH-B2-2025**	PX921704
**13046739-ATH-B1-2025**	PX921705
**13045474-ATH-B2-2025**	PX921706
**13003711-ATH-B2-2025**	PX921707
**13007671-ATH-B2-2025**	PX921708
**13017190-ATH-A2.2.2-2025**	PX921709
**12988320-ATH-B2-2025**	PX921710
**13190785-ATH-B2-2025**	PX921711
**13149309-ATH-B2-2025**	PX921712
**13129850-ATH-B2-2025**	PX921713
**13117137-ATH-A2.2.2-2025**	PX921714
**13057877-ATH-A2.2.2-2025**	PX921715
**13096250-ATH-B2-2025**	PX921716
**13059108-ATH-B2-2025**	PX921717
**12958482-ATH-B2-2025**	PX921718
**13166891-ATH-A2.2.2-2025**	PX921719
**13263871-ATH-A2.2.2-2025**	PX921720
**13157524-ATH-A2.2.2-2025**	PX921721
**13114053-ATH-A2.2.2-2025**	PX921722
**13128399-ATH-A2.2.2-2025**	PX921723
**13135637-ATH-B2-2025**	PX921725
**13011209-ATH-A2.2.2-2025**	PX921728
**13019205-ATH-A2.2.2-2025**	PX921729
**13000461-ATH-A2.2.2-2025**	PX921730
**13049422-ATH-A2.2.2-2025**	PX921731
**13047225-ATH-A2.2.2-2025**	PX921732
**13037961-ATH-A2.2.2-2025**	PX921733
**12986269-ATH-A2.2.2-2025**	PX921734
**13037885-ATH-A2.2.2-2025**	PX921735
**13038499-ATH-B2-2025**	PX921736
**12971374-ATH-B2-2025**	PX921737
**13009177-ATH-B2-2025**	PX921738
**12971387-ATH-B2-2025**	PX921739
**13032880-ATH-B2-2025**	PX921740
**480979-ATH-B2-2025**	PX921741
**13000048-ATH-B2-2025**	PX921742
**13038717-ATH-B2-2025**	PX921743
**13082770-ATH-A2.2.2-2025**	PX921744
**13113586-ATH-A2.2.2-2025**	PX921745
**13146584-ATH-A2.2.2-2025**	PX921746
**13186053-ATH-A2.2.2-2025**	PX921747
**13113030-ATH-B2-2025**	PX921748
**13136314-ATH-B2-2025**	PX921749

**13177694-ATH-B2-2025**	PX921750
**13054911-ATH-B2-2025**	PX921751
**13124785-ATH-B2-2025**	PX921752
**13154459-ATH-B2-2025**	PX921753
**12962923-ATH-B2-2025**	PX921754

Supplementary Table 2. Information about all the strains used for the phylogenetic analyses.

GENBANK ACCESSION NO	GENOTYPE	COUNTRY OF ORIGIN	YEAR OF ISOLATION
**KJ627437**	A2.2.1	PER	2011
**KJ627436**	A2.2.1	PER	2009
**KJ627435**	B1	PER	2009
**KJ627434**	A2.2.1	PER	2008
**KJ627433**	A2.1	PER	2012
**KJ627432**	B2	PER	2009
**KJ627431**	B1	PER	2009
**KJ627430**	A2.2.1	PER	2009
**KJ627429**	A2.2.1	PER	2011
**KJ627428**	A2.1	PER	2012
**KJ627427**	A2.1	PER	2012
**KJ627426**	A2.2.1	PER	2008
**KJ627425**	A2.1	PER	2011
**KJ627424**	A2.2.1	PER	2009
**KJ627423**	A2.1	PER	2012
**KJ627422**	A2.2.1	PER	2009
**KJ627421**	A2.1	PER	2011
**KJ627420**	A2.2.1	PER	2009
**KJ627419**	A2.1	PER	2011
**KJ627418**	A2.1	PER	2011
**KJ627417**	A2.2.1	PER	2010
**KJ627416**	A2.1	PER	2011
**KJ627415**	A2.1	PER	2011
**KJ627414**	B2	PER	2010
**KJ627413**	A2.1	PER	2012
**KJ627412**	A2.2.1	PER	2009
**KJ627411**	A2.2.1	PER	2010
**KJ627410**	A2.2.1	PER	2009
**KJ627409**	A2.2.1	PER	2009
**KJ627408**	A2.2.1	PER	2010
**KJ627407**	A2.1	PER	2011
**KJ627406**	A2.2.1	PER	2010
**KJ627405**	A2.2.1	PER	2010
**KJ627404**	A2.2.1	PER	2008
**KJ627403**	A2.2.1	PER	2009
**KJ627402**	A2.2.1	PER	2009
**KJ627401**	A2.2.1	PER	2011
**KJ627400**	B2	PER	2010
**KJ627399**	A2.2.1	PER	2010

**KJ627398**	A2.1	PER	2011
**KJ627397**	B2	PER	2010
**KJ627396**	A2.2.1	PER	2010
**KJ627394**	A2.1	PER	2011
**KF530179**	B1	AUS	2003
**KF530178**	B2	AUS	2004
**KF530173**	B1	AUS	2004
**KF530171**	B1	AUS	2004
**KF530167**	B1	AUS	2004
**KF530164**	B1	AUS	2004
**KF530163**	B1	AUS	2004
**MK820375**	B1	CHN	2018
**OM262418**	B2	CHN	2020
**OM262417**	A2.2.2	CHN	2018
**OM262416**	A2.2.2	CHN	2019
**OM262415**	A2.2.2	HN	2019
**OM262414**	A2.2.2	CHN	2019
**OM262412**	A2.2.2	CHN	2018
**OM262411**	A2.2.2	CHN	2018
**OM262410**	A2.2.2	CHN	2018
**OM262409**	A2.2.2	CHN	2017
**OM262408**	A2.2.2	CHN	2017
**OM262407**	A2.2.2	CHN	2018
**OM262405**	A2.2.2	CHN	2017
**OM262404**	A2.2.2	CHN	2018
**OM262396**	A2.2.2	CHN	2019
**OM262395**	A2.2.2	CHN	2019
**MK588636**	B1	KEN	2012
**MK588635**	A2.1	ZAM	2012
**MK087726**	A2.2.2	CHN	2018
**OL794387**	B2	NL	2017
**OL794386**	A2.2.2	NL	2016
**OL794385**	B2	NL	2006
**OL794384**	A2.1	NL	2006
**OL794383**	B1	NL	2017
**OL794381**	B2	NL	2006
**OL794380**	B2	NL	2016
**OL794379**	B2	NL	2006
**OL794378**	A2.2.2	NL	2017
**OL794377**	B2	NL	2016
**OL794376**	B2	NL	2006
**OL794375**	A2.2.2	NL	2017
**OL794374**	A2.2.2	NL	2016
**OL794373**	A2.2.1	NL	2015

**OL794372**	B1	NL	2014
**OL794371**	A2.2.2	NL	2011
**OL794370**	A2.1	NL	2010
**OL794369**	A2.2.1	NL	2006
**OL794368**	B2	NL	2019
**OL794367**	A2.2.1	NL	2016
**OL794366**	A2.2.1	NL	2015
**OL794365**	B2	NL	2014
**OL794364**	A2.2.2	NL	2013
**OL794363**	A2.1	NL	2012
**OL794362**	A2.1	NL	2010
**OL794361**	A2.2.1	NL	2009
**OL794360**	A2.2.2	NL	2008
**OL794359**	A2.2.1	NL	2007
**OL794358**	B1	NL	2006
**OL794357**	B1	NL	2005
**OL794356**	B1	NL	2003
**OL794355**	A1	NL	2002
**OK644703**	B1	CHN	2020
**MN867464**	A2.2.2	CHN	2009
**MK588637**	B2	KEN	2012
**MH150889**	A2.1	NL	2017
**MH150888**	A2.1	NL	2017
**MG431250**	B2	BRA	2010
**KY474545**	A2.2.2	USA	2016
**KY474542**	A2.2.2	USA	2016
**KY474541**	A2.2.2	USA	2016
**KY474539**	A2.2.2	USA	2016
**KY474530**	A2.2.1	USA	2016
**KF516922**	B1	KOR	2011
**JN184402**	B2	USA	1999
**JN184401**	B1	USA	1998
**JN184400**	A2.1	USA	1994
**JN184399**	A1	USA	1999
**HM197719**	B2	USA	2008
**DQ843659**	A2.2.1	CHN	2006
**DQ843658**	B2	CHN	2006
**EF535506**	B2	TWN	1999
**GQ153651**	A2.2.2	CHN	2008
**AY525843**	B1	NL	1999
**PV218189**	B1	CHN	2017
**PV218188**	B2	CHN	2017
**PV218187**	A2.2.2	CHN	2015
**PV218186**	B1	CHN	2017

**PV218185**	B1	CHN	2017
**PV218178**	A2.2.2	CHN	2015
**PV218172**	A2.2.1	CHN	2017
**PV218166**	A2.2.2	CHN	2015
**PV218165**	B2	CHN	2017
**PV218164**	B1	CHN	2017
**PV218163**	B1	CHN	2017
**PV218162**	A2.2.2	CHN	2017
**PV218157**	A2.2.2	CHN	2016
**PV218155**	B1	CHN	2016
**PV218154**	A2.2.2	CHN	2015
**PV218153**	A2.2.2	CHN	2016
**PV218152**	B1	CHN	2016
**PV218148**	B1	CHN	2016
**PV218142**	A2.2.2	CHN	2015
**PV218134**	B1	CHN	2015
**PV218130**	B1	CHN	2015
**PV218124**	B1	CHN	2015
**PV218121**	A2.2.2	CHN	2014
**PV218118**	A2.2.2	CHN	2015
**PV218117**	B2	CHN	2024
**PV218116**	B2	CHN	2024
**PV218115**	A2.2.2	CHN	2024
**PV218114**	B2	CHN	2024
**PV218113**	B2	CHN	2024
**PV218112**	A2.2.2	CHN	2015
**PV218111**	B2	CHN	2024
**PV218099**	B2	CHN	2024
**PV218059**	A2.2.2	CHN	2014
**PV218058**	B2	CHN	2024
**PV218052**	B2	CHN	2024
**PV218049**	A2.2.2	CHN	2015
**PV218048**	B2	CHN	2024
**PV218010**	A2.2.2	CHN	2024
**PV217981**	A2.2.2	CHN	2023
**PV217980**	A2.2.2	CHN	2023
**PV217979**	A2.2.2	CHN	2023
**PV217978**	A2.2.2	CHN	2023
**PV217977**	A2.2.1	CHN	2023
**PV217976**	A2.2.2	CHN	2023
**PV217975**	A2.2.2	CHN	2023
**PV217974**	A2.2.2	CHN	2015
**PV217973**	A2.2.2	CHN	2023
**PV217972**	A2.2.1	CHN	2023

**PV217967**	A2.2.1	CHN	2023
**PV217966**	A2.2.2	CHN	2023
**PV217961**	A2.2.2	CHN	2022
**PV217959**	B2	CHN	2021
**PV217958**	A2.2.2	CHN	2022
**PV217957**	B2	CHN	2021
**PV217956**	A2.2.2	CHN	2022
**PV217955**	B2	CHN	2020
**PV217954**	B2	CHN	2020
**PV217953**	B2	CHN	2020
**PV217952**	B2	CHN	2020
**PV217951**	A2.2.2	CHN	2014
**PV217950**	B2	CHN	2020
**PV217949**	A2.2.2	CHN	2015
**PV217948**	B2	CHN	2019
**PV217946**	B2	CHN	2019
**PV217945**	B2	CHN	2019
**PV217944**	A2.2.2	CHN	2019
**PV217943**	B2	CHN	2019
**PV217903**	A2.2.2	CHN	2018
**PV217902**	A2.2.2	CHN	2018
**PV217901**	B2	CHN	2018
**PV217887**	B2	CHN	2015
**PV217852**	B1	CHN	2017
**PX120322**	A2.2.1	KEN	2024
**PX120319**	A2.2.2	KEN	2024
**PX120317**	A2.2.2	KEN	2023
**PX120316**	B1	KEN	2021
**PV660309**	A2.2.2	USA	2023
**PV660308**	B2	USA	2023
**PV081664**	B2	BRA	2024
**PV081662**	A2.2.2	BRA	2024
**PQ634956**	A2.2.2	USA	2022
**PQ634937**	A2.2.2	USA	2022
**PQ634896**	A2.2.2	USA	2021
**PQ523787**	A2.2.2	USA	2023
**PP947664**	B1	USA	2022
**PP947663**	B2	USA	2019
**PP947662**	B1	USA	2019
**PP947654**	A2.2.2	USA	2019
**PP947653**	B1	USA	2019
**PP947652**	B1	USA	2018
**PP947651**	A2.2.2	USA	2020
**PP947650**	A2.2.2	USA	2020

**PP947649**	A2.2.2	USA	2019
**PV217873**	A2.2.2	CHN	2015
**PV217863**	A2.2.2	CHN	2018
**PV217862**	A2.2.2	CHN	2015
**PV217858**	A2.2.2	CHN	2018
**PV217857**	B1	CHN	2018
**PV217856**	A2.2.2	CHN	2018
**PV217855**	A2.2.2	CHN	2018
**PV217854**	A2.2.2	CHN	2018
**PV217851**	A2.2.2	CHN	2015
**PV217843**	B1	CHN	2017
**PV217842**	A2.2.2	CHN	2017
**PX120323**	A2.2.1	KEN	2024
**PX120321**	A2.2.1	KEN	2024
**PX120320**	A2.2.2	KEN	2023
**PX120318**	A2.2.2	KEN	2023
**PV660311**	B2	USA	2023
**PV660310**	B1	USA	2023
**PV660307**	A2.2.2	USA	2023
**PV660306**	A2.2.2	USA	2024
**PV081667**	B2	BRA	2024
**PV081666**	A2.2.2	BRA	2024
**PV081665**	A2.2.1	BRA	2024
**PV081663**	B2	BRA	2024
**PQ634888**	A2.2.2	USA	2019
**PQ634884**	A2.2.2	USA	2020
**PP947639**	B2	USA	2022
**PP947638**	A2.2.2	USA	2019
**PP947637**	B2	USA	2019
**PP947632**	A2.2.2	USA	2021
**PP947580**	B2	USA	2019
**PP947579**	B2	USA	2020
**PP947578**	B2	USA	2019
**PP947577**	B2	USA	2020
**PP947576**	B2	USA	2019
**PP947575**	A2.2.2	USA	2019
**PP947574**	A2.2.2	USA	2020
**PP947573**	B1	USA	2019
**PP947572**	B1	USA	2020
**PP947570**	B2	USA	2020
**PP947569**	B1	USA	2020
**PP947568**	B1	USA	2020
**PP947567**	B1	USA	2020
**PP947566**	B2	USA	2019

**PP947565**	B2	USA	2020
**PP947563**	A2.2.2	USA	2020
**PP947562**	A2.2.2	USA	2019
**PP947561**	B2	USA	2019
**PP947558**	B2	USA	2020
**PP947556**	A2.2.2	USA	2020
**PP947555**	B1	USA	2020
**PP947554**	B2	USA	2019
**PP947553**	A2.2.2	USA	2020
**PP315926**	B1	CHN	2023
**MK167040**	A2.2.2	USA	2017
**MK167039**	A2.2.2	USA	2017
**PP716096**	A2.2.2	RUS	2023
**PP716095**	A2.2.2	RUS	2023
**PP716094**	A2.2.2	RUS	2023
**PP716093**	A2.2.2	RUS	2023
**PP591754**	A2.2.2	USA	2023
**PP591753**	A2.2.2	USA	2023
**OR338754**	A2.2.2	CHN	2012
**OR338753**	B1	CHN	2011
**OP345077**	A2.2.2	CHN	2018
**OP345076**	A2.2.2	CHN	2018
**MZ221204**	B2	CHN	2014
**MZ221203**	B2	CHN	2014
**MZ221202**	B1	CHN	2018
**MZ221201**	B1	CHN	2014
**MZ221200**	B1	CHN	2014
**ON168639**	B1	CHN	2017
**MZ504967**	A2.2.2	CHN	2015
**MZ504966**	B2	CHN	2015
**MZ504965**	A2.2.2	CHN	2015
**MZ504964**	B2	CHN	2014
**MZ504963**	B1	CHN	2013
**MZ504962**	A2.2.2	CHN	2013
**MZ504961**	A2.2.2	CHN	2013
**MZ504960**	B1	CHN	2012
**MZ504959**	B1	CHN	2011
**MZ504958**	A2.2.2	CHN	2011
**MZ851993**	A2.2.2	CHN	2018
**MZ851992**	A2.2.2	CHN	2018
**MN745086**	A2.2.2	CHN	2017
**MN745085**	A2.2.2	CHN	2017
**MN745084**	A2.2.2	CHN	2017
**KU821121**	A1	CHN	2012

**FJ168779**	A2.1	NL	2000
**FJ168778**	B2	NL	1994
**LC889311**	B2	JPN	2024
**LC889310**	B2	JPN	2024
**LC889309**	A2.2.2	JPN	2024
**LC889308**	A2.2.2	JPN	2024
**LC889307**	A2.2.2	JPN	2024
**LC889306**	A2.2.2	JPN	2024
**LC817386**	B2	JPN	2022
**LC817385**	B2	JPN	2023
**LC817384**	B1	JPN	2022
**LC823195**	A2.2.2	JPN	2023
**LC82319**	A2.2.2	JPN	2023
**LC789936**	A2.2.2	GABON	2020
**PV178633**	A2.2.2	USA	2024
**PV178565**	A2.2.2	USA	2024
**PV178527**	A2.2.1	USA	2024
**PV178512**	A2.2.2	USA	2023
**PV178427**	B2	USA	2024
**PV178241**	A2.2.2	USA	2024
**PV178224**	A2.2.2	USA	2024
**PV353978**	B1	PER	2022
**PV353977**	A2.2.2	PER	2023
**PV353976**	A2.2.2	PER	2023
**PV353975**	A2.2.2	PER	2023
**PV353974**	A2.2.2	PER	2023
**PV353973**	A2.2.2	PER	2023
**PV353972**	A2.2.2	PER	2023
**PV353971**	A2.2.2	PER	2023
**PV353970**	A2.2.2	PER	2022
**PV353969**	A2.2.2	PER	2024
**LC769218**	B1	JPN	2021
**LC769217**	B2	JPN	2022
**LC769210**	B1	JPN	2018
**LC769219**	A2.2.2	JPN	2019
**LC769216**	A2.2.2	JPN	2019
**LC769215**	A2.2.2	JPN	2019
**LC769214**	A2.2.2	JPN	2019
**LC769213**	A2.2.2	JPN	2019
**LC769212**	B1	JPN	2018
**LC769211**	B1	JPN	2018
**LC769209**	A2.2.2	JPN	2019
**LC769208**	A2.2.2	JPN	2018
**LC756671**	B1	JPN	2018

Supplementary Table 3. Monthly total tests performed by multiplex syndromic molecular assays and the respective number of total and hMPV-positive samples for 2024 and 2025.

Month	*Total FA Tests 2024*	*Total hMPV Positive 2024*	*hMPV Positivity Rates 2024*	*Total FA Tests 2025*	*Total hMPV Positive 2025*	*hMPV Positivity Rates 2025*
January	91	1	1,099	693	13	1,88
February	234	1	0,43	337	18	5,34
March	375	17	4,53	77	16	20,78
April	396	26	6,57	75	12	16
May	405	18	4,44	100	2	2
June	348	4	1,15	79	0	0
July	347	1	0,29	93	0	0
August	347	0	0	91	0	0
September	335	0	0	89	0	0
October	386	0	0	146	0	0
November	271	0	0	118	0	0
December	501	4	0,8	180	1	0,55

Supplementary Table 4. Co-detection rates of other respiratory pathogens along with hMPV in 2024 and 2025.

Year	hMPV only	hMPV + other pathogen	Total
**2024**	61 (84,7%)	11 (15,3%)	72
**2025**	50 (80,64%)	12 (19.36%)	62

Supplementary Table 5. Age distribution of hMPV-positive patients in 2024 and 2025.

Age Group	2024	2025
**0–4**	18 (25%)	9 (14,5%)
**5–17**	16 (22,2%)	10 (16,12%)
**18–64**	18 (25%)	21 (33,9%)
**≥65**	20 (27.8%)	22 (35,5%)
